# Progress and Challenges of Amniotic Fluid Derived Stem Cells in Therapy of Ischemic Heart Disease

**DOI:** 10.3390/ijms22010102

**Published:** 2020-12-24

**Authors:** Yi-Hsien Fang, Saprina P. H. Wang, Hsien-Yuan Chang, Pei-Jung Yang, Ping-Yen Liu, Yen-Wen Liu

**Affiliations:** 1 Institute of Clinical Medicine, College of Medicine, National Cheng Kung University, Tainan 70457, Taiwan; eddiefang0023@gmail.com (Y.-H.F.); saprina15@gmail.com (S.P.H.W.); Doyeric0926@yahoo.com (H.-Y.C.); ybrpooh719@gmail.com (P.-J.Y.); larry@mail.ncku.edu.tw (P.-Y.L.); 2 Division of Cardiology, Department of Internal Medicine, National Cheng Kung University Hospital, College of Medicine, National Cheng Kung University, Tainan 70403, Taiwan; 3 Center of Cell therapy, National Cheng Kung University Hospital, College of Medicine, National Cheng Kung University, Tainan 70403, Taiwan

**Keywords:** amniotic fluid stem cells, pluripotent stem cells, stem cell therapy, cardiovascular diseases, regenerative therapy

## Abstract

Cardiovascular disease is the leading cause of deaths worldwide, claiming an estimated total of 17.9 million lives each year, of which one-third of the people are under the age of 70 years. Since adult cardiomyocytes fail to regenerate, the heart loses the ability to repair itself after an injury, making patients with heart disease suffer from poor prognosis. Pluripotent stem cells have the ability to differentiate into cardiomyocytes in vitro through a well-established process, which is a new advancement in cardiac regeneration therapy. However, pluripotent stem cell-derived cardiomyocytes have certain drawbacks, such as the risk of arrhythmia and immune incompatibility. Thus, amniotic fluid stem cells (AFSCs), a relatively novel source of stem cells, have been exploited for their ability of pluripotent differentiation. In addition, since AFSCs are weakly positive for the major histocompatibility class II molecules, they may have high immune tolerance. In summary, the possibility of development of cardiomyocytes from AFSCs, as well as their transplantation in host cells to produce mechanical contraction, has been discussed. Thus, this review article highlights the progress of AFSC therapy and its application in the treatment of heart diseases in recent years.

## 1. Introduction

Despite huge advances in medical therapy nowadays, cardiovascular diseases are still the leading cause of mortality worldwide. Moreover, there is an upward trend in mortality every year. Although novel pharmacological therapeutics and surgical or percutaneous transluminal intervention have been developed in the recent years, however, the prognosis of terminal stage heart failure or severe ischemic heart disease is worse than many malignancies [1]. It could be because these therapies cannot lead to cardiac regeneration. The heart is composed of cardiomyocytes that possess varying regenerative abilities at different stages of development in mammals. During the fetal period, the cardiomyocytes undergo a complete cell cycle, but they lose their ability to divide within a few days after birth. The cardiomyocytes of adult mammals are terminally differentiated cells with a rate of regeneration of only less than 1% per year [2].

When the adult heart is injured, it enters an incomplete cell cycle but not complete cell division, resulting in hypertrophy of the cardiomyocytes. If necrosis of the myocardium occurs, the cardiomyocytes lose their intrinsic regenerative ability, leading to myocardial fibrosis, poor cardiac contraction, and poor prognosis in patients with ischemic heart disease [2,3]. Thus, the compensatory effect increases the burden on the heart, posing a high risk of its failure [4]. The most effective treatment for heart failure is heart transplantation, but due to a shortage in the supply of donor hearts, only a few patients undergo this treatment. Therefore, use of stem cells to replace the necrotic cardiomyocytes is gaining momentum in the research area of heart regeneration.

## 2. Advantages and Limitations of Different Types of Stem Cells in Cardiac Regeneration

There are different types of stem cells involved in the development process of organisms. Based on differentiation ability, stem cells are categorized as totipotent, pluripotent, multipotent, and unipotent. Among these categories, embryonic stem cells (ESCs) are pluripotent in nature, which can be induced to differentiate into almost every cell type; however, their application is limited due to ethical concerns [5].

Compared with ESCs, multipotent stem cells are located at multiple sites, such as adipose tissue, connective tissue, bone marrow, etc., and most of them are classified as mesenchymal stem cells (MSC). MSCs are considered to have immune privileges in regenerative therapy. They secrete many biologically active molecules, including cytokines, growth factors, and chemokines and regulate the activity of immune cells such as B cells, T cells, dendritic cells (DC), natural killer (NK) cells, neutrophils, and macrophages through autocrine and paracrine effects [5,6]. MSC are also not restricted by ethics and are found in many cell types, since they can differentiate into some specific types of cells. Moreover, most MSCs have a limited ability of cardiomyocyte differentiation [6,7,8]. In addition, using MSC as a material of myocardial repair has low efficacy. After using MSC derived cardiomyocyte after myocardial infarctions in animal models, the function of the left ventricular still has not been significantly improved [7].

In 2007, Shinya Yamanaka obtained artificial pluripotent stem cells by transfecting four transcription factors into somatic cells; these reprogrammed cells were called induced pluripotent stem cells (iPSCs) [8]. Recently, iPSCs have been used in a variety of preclinical research on tissue engineering and there have been breakthroughs in research on regenerative medicine. However, it is expensive to use autologous cells and reprogram them into iPSCs; meanwhile, allogeneic iPSCs may cause immune rejection. The limitations mentioned above are the obstacles in the clinical applications of iPSCs. Recently, many studies established a protocol for the differentiation of cardiomyocytes from pluripotent stem cells in vitro, including simulation of the stem cells using small-molecule drugs, such as vascular endothelial growth factor and Wnt signal inhibitor/activator, [9] which allow the stem cells to initially progress from embryonic stage to mesoderm and finally to cardiomyocytes. The other strategy is the addition of growth factors, such as fibroblast growth factor 2 (FGF2), transforming growth factor β (TGF-β), activin A, and bone morphogenetic protein 4 (BMP4), which are important for the cardiovascular development [10]. Up to 90% differentiation ability of the cardiomyocytes derived from pluripotent stem cells have confirmed and identified by flow cytometry and spontaneous contraction occurs. Cardiomyocytes differentiated from embryonic stem cells (ESC-CMs) also show good therapeutic effects in animal models of myocardial infarction. The overall characteristics comparison of each types stem cells is organized in Table 1.

## 3. Therapeutic Effect of Human Pluripotent Stem Cell-Derived Cardiomyocytes (PSC-CMs) on Myocardial Infarction

Recently, several large animal studies proved that PSC-CMs transplantation in the infarcted myocardium could restored the post-MI cardiac systolic function. The transplanted PSC-CMs could survive in the recipient animals for more than three months, leading to improvement of cardiac contractility and LV systolic function. Additionally, the graft also exhibited regular calcium transients, which were synchronized with the hosts’ cardiomyocytes, indicating electromechanical coupling between the graft and host tissue. At present, the common method of cardiac regeneration therapy by using stem cell-derived cardiomyocytes is intramyocardial cell injection; this method has the best therapeutic effect and the graft can replace the necrotic myocardial tissue and perform synchronous contraction with host myocardium, and it can maintain long-term curative effect [20,22]. The ejection fraction dramatically increased by approximately more than 10% after the transplantation of human ESC-CMs in a non-human primate model of myocardial infarction [20,22]. The use of stem cell-derived cardiomyocyte formed-patches to attach the outer wall of the heart is also one of the transplantation methods. Although the ventricular contractility has temporarily improved after transplantation, long-term effects cannot be seen and the host’s necrotic cardiomyocytes cannot be regenerated [23,24], since the heart is a highly dynamic organ, which can make it challenging to anchor the in vitro differentiated-cardiomyocytes to the affected area. Therefore, direct intramyocardial injection of graft cells may be more straightforward than intravenous injection or cell patches. 

Another limitation is the immune response, since xenogeneic transplantation definitely resulted in graft rejection. Therefore, strong immunosuppressive agents have to be administered to diminish the post-transplant immune reaction. Nevertheless, such a combination of potent immunosuppressants would result in severe adverse events, such as fatal infection, malignancy, kidney injury, etc. Thus, those stem cells which have immune privilege may be the answer to this obstacle. Mismatch of major histocompatibility antigens (MHC) or human leukocyte antigens (HLA) specific to human genetics is the main cause of allograft rejection. MHC class I and class II proteins express on the cell surface and can be recognized by T lymphocytes and the function of non-classical class III of MHC remain unknown. In the human genetic locus, MHC contains more than 20,000 alleles, resulting in individual differences [25]. According to the above, autologous transplantation is an ideal way to avoid rejection. Due to the homozygote property, the advantage of using autologous iPSC, MHC-matched transplantation evades the immune response, which reduces macrophage and cytokine attacks. However, due to the high cost and long preparation time, using iPSC-derived self-transplantation cells may be difficult to apply to acute symptoms such as myocardial infarction. Moreover, autologous iPSCs are limited by disease-specific anomaly or genetic defects during reprograming; therefore, allogeneic transplantation is the priority selection [26].

It is well known that MHC class I related molecules HLA-A, -B, and -C are recognized by CD8 positive T cells. MHC class II is responsible for encoding HLA-DRA, -DRB1, -DRB3, -DRB4, -DRB5, -DQA1, -DQB1, -DPA1, and -DPB1 and identified by CD4-positive T cells [26]. Therefore, immunosuppressive agents additively eliminate the MHC expression, which is necessary for the rejection reaction.

It has been published that knockout MHC iPSC-derived cardiomyocytes using CRISPR/Cas9 improved the efficiency of the survival time of grafts in vivo. Verification that there is no MHC performance to avoid immune cells attack is necessary. However, the unpredictable problem of gene editing is the limitation in this method [27].

## 4. Characteristics and Application of Amniotic Fluid Derived Stem Cells (AFSCs)

AFSCs may be more primitive than multipotent stem cells because it is indicated that AFSCs can differentiate into three germ layers and specific lineage tissues in vitro [28,29,30]. AFSCs were shown to express CD105, CD73, and CD90, as well as lack the expression of surface molecules, such as CD45, CD34, CD14, CD11b, CD79α, CD19, and HLA-DR, and they have the ability to differentiate into osteoblasts, adipocytes, and chondrocytes in vitro [30,31]. Importantly, there is no ethical concern related to the use of AFSCs because these cells were collected during the routine amniocentesis of second trimester (14–18 weeks of gestation) or cesarean section (end of pregnancy).

In vitro, the primary AFSCs can expand more than 150 times without the shortening of telomerase. In addition, these cells are not tumorigenic after transplantation in animal models [30]. Analysis and comparison of multiple clones of AFSCs revealed that the cells are positive for major histocompatibility (MHC) class I molecules, also called HLA-ABC, but weakly positive for MHC class II. Moreover, AFSCs also express specific embryonic antigens, such as SSEA-4 and CD90 [32,33], that are specific markers in the ESCs, but do not express CD45, CD34, CD133, or other hematopoietic stem cell markers. In addition, more than 90% of the cells express the transcription factor, Oct4. These findings indicate that the differentiation ability of AFSCs lies between multipotent and pluripotent stem cells [32]. These advantages allow AFSCs to be an ideal candidate for use in regenerative medicine.

More detailed analyses show that AFSCs consist of multiple subtypes. Based on different morphologies, AFSCs, collected by amniocentesis scheduled between the 15th and 18th weeks of pregnancy, can be roughly divided into two types of adherent cells—spindle-shaped (SS) and round-shaped (RS) AFSCs, accounting for approximately 6% and 94% of the total cells, respectively. SS-AFSCs show higher ability to proliferate than RS-AFSCs. Both types of AFSCs do not express CD34, CD133, CD31, CD45, CD14, and HLA-DR. However, they express the markers of MSCs, such as CD73, CD105, and CD166, as well as adhesion molecules, such as CD29, CD44, CD49e, and HLA-ABC, as previously described [20]. Selection of AFSCs based on different subtypes can increase their efficiency for preclinical and clinical applications.

## 5. Possibility of Developing Cardiomyocytes from AFSCs

Due to the differentiation ability of AFSCs and low immunogenicity than other allogeneic-transplanted pluripotent stem cells, AFSCs are considered as potential candidates that can compensate for the limitations of pluripotent and multipotent stem cells. It is also known that the development of the mammalian heart is regulated by the Gsk3 and Wnt signaling pathways. Thus, cardiomyocyte differentiation can be induced in vitro from pluripotent stem cells, using small molecule drugs, such as CHIR99021 and IWR1, or recombinant proteins, such as activin and BMP4 [28,29]. It was shown that these differentiated AFSCs had higher levels of α-actin, desmin, and myogenin than the adipose stem cells [21]. Additionally, the cardiac-differentiated AFSCs also expressed the cardiac transcription factor, Nkx2.5, at the metaphase stage of differentiation into cardiomyocytes. A subgroup of the differentiated AFSCs upregulated the expression of proteins, such as cardiac troponins and Connexin 43, which is the main gap-junction protein of cardiomyocytes. This evidence indicated in vitro differentiation of AFSCs along the cardiac lineage. However, spontaneous contraction was not observed at the end of this differentiation process. We analyzed the electrophysiological properties of cardiomyocytes derived from human embryonic stem cells (hESCs) and AFSCs.

Although AFSC expresses specific markers of cardiomyocyte, as the mesenchymal stem cell-derived cardiomyocytes, it cannot contract spontaneously. All the detected action potential came from the cell membrane current of the host cell after transplantation, not autologous [34,35].

We also recorded the spontaneous action currents (AC) and action potentials (APs) in hESC-derived cardiomyocytes (hESC-CM), using the cell current clamp mode but not in AFSC-derived cardiomyocytes (AFSC-CM). In AFSC-CM, the active channels of calcium were also significantly reduced, which led to the lack of spontaneous AC and AP. These results indicated that the well-established Wnt signal-regulated cardiac differentiation protocol is not sufficient to induce the differentiation of AFSCs into functional cardiomyocytes. Therefore, AFSCs may not be a good source to derive cardiomyocytes [31].

In order to solve the problem of poor differentiation efficiency of AFSCs into cardiomyocytes, a strategy was considered to reprogram AFSCs into induced pluripotent stem cells (iPSCs). To test this hypothesis, AFSCs were exposed to the reprogramming factors, including Oct4, Klf4, Sox2, and c-Myc, and their growth into iPSC colonies was monitored. After the cardiomyocyte differentiation process, CM markers in the early stage of differentiation, such as ISL1 and Nks2.5, and those in the late stage of differentiation, such as TNNT2 and Connexin 43, were expressed in AFSC- iPSC and cell spontaneity was observed [36,37]. The overall characterization of cardiomyocyte differentiation is organized in Table 2. The comparison of ion currents of in vitro derived cardiomyocyte also organized in Table 3. Considering the advantage of immune tolerance of AFSCs, we investigated whether the AFSC-iPSC-derived cardiomyocytes (AFSC-iPSC-CM) also maintained immune properties as the primary AFSCs.

It was confirmed that the AFSCs can be reprogrammed into iPSCs, which can differentiate into cardiomyocytes that express MHC class I and lack MHC class II molecules. They also possess the specific electrophysiological properties and spontaneous contraction of cardiomyocytes. Transplanted AFSC-iPSC-CMs survived in the infarcted area of the rat heart and restored heart functions after myocardial infarction. Thus, AFSC-iPSC-CMs are potential therapeutic products for the treatment of ischemic heart disease [21]. It was reported that intramyocardial transplantation of AFSCs in the infarcted rat hearts would improve post-infarct cardiac function, but only a small number of the transplanted AFSCs could survive in the hearts and differentiate into endothelial cells and smooth muscle cells [53]. Moreover, these transplanted AFSCs minimally differentiated into cardiomyocyte. Thus, apart from differentiating into cardiomyocytes, AFSCs also provide mechanical contractility to the host heart and may also repair the injured hearts through paracrine signaling [53,54,55]. Furthermore, how to diminish post-transplant rejection in an allogeneic or xenogeneic environment should be a key issue of successful allogeneic stem cell therapy.

## 6. Paracrine Regulation of Amniotic Fluid Stem Cells

In addition to pluripotent characteristics and immune privileges, AFSCs showed unique paracrine properties in the preclinical models of several diseases. AFSCs can secrete vesicles through automatic paracrine signaling or via stimulation. Vesicles secreted by AFSCs may also contain micro RNA. It has been confirmed that these vesicles can reduce the H_2_O_2-_induced oxidative stress in vitro and reduce cell apoptosis through the enrichment of exosomes containing regulatory microRNA. The immune tolerance and regulation capabilities of AFSCs were evaluated through peripheral blood mononuclear cell (PBMC) co-culture in vitro; AFSC inhibited the activation of CD19/CD27 positive B cells and reduced the inflammation of skeletal muscles in vivo. In addition, AFSCs exhibited the ability to generate vascular endothelial cells [55].

Although the paracrine effect of AFSCs has been studied, its complex mechanism is still unclear. An in vitro identification of AFSCs also expressed high IDO1. IDO1 is the enzyme which was stimulated by the early immune response marker-interferon-γ; it goes through para-vesicles, resulting in arrested T cell proliferation, induced apoptosis of effect T cells, and active FOXP3/CD25 positive regular T cells, which regulate immune response. Overall, the action of IDO1, through paracrine signaling, contributes to immune regulation and does not require cell contact [56]. Among different AFSC subgroups, SS-AFSCs show greater differentiation and protective effects. Therefore, SS-AFSCs may have potential clinical applications in the treatment of myocardial infarction. In a rat model of myocardial infarction, it was shown that an intravenous administration of SS-AFSC vesicles had cardioprotective effects, which reduced the infarct size by approximately 27% and also promoted the migration of endothelial cells, resulting in increased angiogenesis. These effects may have arisen from proteins embedded in the AFSC vesicles, such as PTX3, MIF, SDF1, and BGN, as well as other chemotactic factors [57]. Auto-secretion of vesicles from AFSCs has significant anti-inflammatory effects and promotes angiogenesis after transplantation to the animal heart, which may be the underlying mechanism of AFSCs to promote muscle regeneration after heart injury [58].

## 7. Unmet Needs before Clinical Trial of Amniotic Fluid Stem Cell-iPSC-CMs in Myocardial Regeneration

### 7.1. Genetic Inheritance of Donor

Despite AFSC being predominant on immunodominance and its ability to differentiate into functional cardiomyocytes after being reprogrammed into iPSC, before entering the clinical trial, there remained some restrictions. Reprogramming autologous cells to iPSC may increase the risk on genetic mutations. The majority of mutations were missense (66%), nonsense (4%), or splice variants (3%). Additionally, there was the generation of teratoma [59,60]. In the in vivo study, it also showed that in the offspring mice from somatic cell nuclear transfer (SCNT), the single-nucleotide variation analysis demonstrated that the number of point gene mutations will increase in the new generation, and when breeding exceeded the sixth generation, the offspring mice’s survival rate may be affected [61].

### 7.2. Tumorigenesis

Process of iPSC production may generate teratomas which is inevitable and currently is unresolved. During the process of somatic cell reprogramming, the proto-oncogene c-Myc significantly promoted the generation of iPSC, but also increased tumor formation in iPSC-derived chimeric mice [62]. In the initial stage of the genome reprogramming process, the G1/S cell cycle checkpoint showed defects in a short-lived manner. CDK2 and cyclin CDK4/6 were massively manifested to accelerate the G1/S phase [63]. The phenotypes are similar to cancer cells [64]. Supposing the undifferentiated iPSC remains in the iPSC-derived cardiomyocytes, it may increase the risk of potential carcinogens, which depends on the different cell line characterization [65].

### 7.3. Arrhythmia

Normal cardiomyocytes in each adult independent individual is well-differentiated, which is the restriction on iPSC- or ESC-derived cardiomyocytes. Immature cardiomyocyte transplantation caused arrhythmia, including premature ventricular contractions, couplets, triplets, and non-sustained ventricular tachycardia and incomplete ventricular electrophysiology [66,67]. Moreover, compared with mature cardiomyocytes, iPSC-CM shows lower negative resting membrane potential (RMP) [68] and immature action potential, similar to fetal phenotype.

## 8. Conclusions and Future Directions

**Figure 1:**
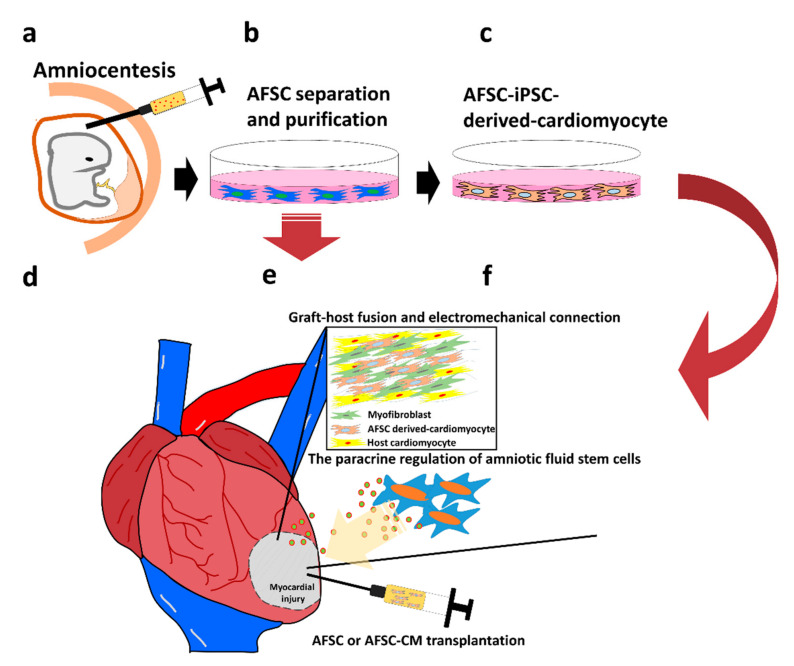
The mechanism of myocardial regeneration by using amniotic fluid stem cells. **a.** Amniotic fluid was taken out during pregnancy by amniocentesis and amniotic fluid stem cells were isolated and purified. **b.** After specific screening, amniotic fluid stem cells were amplified in vitro for use directly or **c.** induced into cardiomyocyte through in vitro well established differentiation procedures. **d.** Via intramyocardial injection to transplants amniotic fluid stem cells or amniotic fluid stem cells derived cardiomyocytes into ischemic necrotic myocardial tissue. The injuried myocardium were repaired by **e.** graft fusion with host myocardium, and act coupling contraction and current conduction. **f.** Amniotic fluid stem cells may also protection cardiomyocytes through paracrine by release growth factors into myocardial microenvironmental.

Significant progress has been made in cardiac regenerative medicine and engineering by using AFSCs as an alternative source of stem cells. Although the extensive preclinical studies conducted on AFSCs support their protective and repair effects, their exact mechanism of action is still not fully understood. We described the two major roles of stem cells in myocardial regeneration, including cardiomyocyte differentiation, as well as transplantation in host cells to produce mechanical contraction and paracrine activation of graft cells to induce endogenous repair mechanisms. We conclude the mechanism of myocardial regeneration by using amniotic fluid stem cells in Figure 1. However, before entering clinical trials, the risk of post PSC-CM transplantation ventricular arrhythmia still remains a major concern; in addition, many secreted factors and interaction between cells have yet to be determined. It is worth noting that the unique immunological characteristics of AFSCs support their clinical applications. Pre-clinical trials have shown promising signs of therapeutic effects of AFSCs in animal models with heart disease, providing solutions for the limitations that exist in the treatment of heart diseases. Thus, in the future, it may so happen that allogeneic AFSCs will be used as commercially available medical products.

## Figures and Tables

**Table 1:** Characteristics comparison of each types stem cells.

Type	Embryonic Stem Cells (ESC)	Mesenchymal Stem Cells (MSC)	Induced Pluripotent Stem Cells (iPSC)
Cell source	Inner cell mass of mulberry embryo [11]	Skin, fat, cord blood, amniotic membrane, bone marrow [12]	Somatic cell [8]
Plasticity	Pluripotent [11]	Multipotent [13]	Pluripotent [8]
Tumorization	Yes [14,15]	No [16,17,18]	Yes [19]
Ethical restriction	Yes	No	No
Functional cardiomyocyte differentiation ability	Yes [20]	No [7]	Yes [21]

**Table 2:** Characterization of cardiomyocyte differentiation.

Type	ESC-CM	MSC-CM	iPSC-CM	AFSC-CM	AFSC-iPSC-CM
cTnT expression	Yes [22]	Yes [38,39]	Yes [40]	Yes [41]	Yes [41]
Contractility	Yes [22]	No [7]	Yes [42]	No [41]	Yes [41]
Therapeutic effect of myocardial infarction in animal	Yes [20]	Restrictive [43]	Yes [44]	Restrictive [45]	Yes [21]

**Table 3:** Comparison of ion currents of in vitro derived cardiomyocyte.

Type	ESC-CM	MSC-CM	AFSC-CM	AFSC-iPSC-CM
Ca2 + cycling	YES [46,47]	No [48]	YES [41]	YES [21]
*I*_na_ Nav1.5	YES [49]	Unreported	YES [50]	YES [21]
*I*_Ca_ Cav1.2	YES [49]	Unreported	YES [50]	YES [21]
*I*_ks_ Kv7.1	YES [51]	Unreported	YES [52]	Unreported

## Data Availability

We cited the data of the original published articles, organized and classify them from an objective perspective.

## Abbreviations

AFSCs	amniotic fluid stem cells
ESCs	embryonic stem cells
iPSCs	induced pluripotent stem cells
FGF2	fibroblast growth factor 2
TGF-β	transforming growth factor β
BMP4	bone morphogenetic protein 4
ESC-CMs	cardiomyocytes differentiated from embryonic stem cells
hESCs	human embryonic stem cells
hESC-CM	hESC-derived cardiomyocytes
AFSC-CM	AFSC-derived cardiomyocytes
